# The Current Practice of Assisted Hatching for Embryos in Fertility Centres: a General Survey

**DOI:** 10.1007/s43032-022-00931-0

**Published:** 2022-04-11

**Authors:** Yaqiong Liu, Celine Jones, Kevin Coward

**Affiliations:** 1 Nuffield Department of Women’s and Reproductive Health, Women’s Centre, University of Oxford, John Radcliffe Hospital, Level 3, Headington, Oxford, OX3 9DU UK; 2 Present address: Centre for Gene Therapy and Regenerative Medicine, King’s College London, Guy’s Hospital, 28th Floor, Tower Wing, London, SE1 1UL UK

**Keywords:** Assisted hatching, Survey, Fertility centre, Laser-assisted, Zona pellucida, Embryo biopsy, Embryo transfer

## Abstract

At present, there is no standardised protocol for assisted hatching (AH) and the field is beset with contradictory data. We hypothesised that such contradiction may be related to inconsistencies in clinical practice. This study aimed to investigate the application, preferences, and variations of AH in current clinical practice prior to embryo transfer (AHpET) and biopsy (AHpBP). An online voluntary survey, consisted of 25 questions regarding different aspects of AH, was circulated amongst different fertility centres via newsletters between October 2019 and March 2020. One-hundred twenty-nine different fertility centres participated in the survey. AHpBP was widely used (90.6% [48/53]) amongst these centres, especially for trophectoderm biopsy (92.2% [47/51]). In contrast, only 64.6% (73/113) of centres administrated AHpET; the application of AHpET was even lower in UK-based centres (36.6% [15/41]). Although laser pulses have become the predominant technique for AH, significant variation existed in the precise strategy. Zona pellucida (ZP) drilling was the main method for AHpBP, whilst both ZP drilling and ZP thinning were applied equally for AHpET. Furthermore, the ZP manipulation varied widely with regards to the size of the ZP opening and the extension of ZP thinning. This is the first representative survey relating to the current practice of AH. Laser-assisted AH is used extensively, especially for AHpBP. However, there is significant disparity in clinical practice across different centres. Future research should aim to create a standardised protocol for AH to help reduce the evident variation in clinical practice and investigate the true value of AH.

**Supplementary Information:**

The online version contains supplementary material available at 10.1007/s43032-022-00931-0.

## Introduction

The past 40 years have witnessed a significant development in the field of assisted reproductive technology (ART), with many different techniques having undergone major improvement. Although the success rates of ART continue to increase, some ART-derived embryos still fail to undergo successful implantation. To achieve a successful pregnancy, the embryo needs to escape from the zona pellucida (ZP), an outer glycoprotein coat, prior to implantation into the uterus. This physiological process is referred to as ‘hatching.’ Failure to hatch, due to abnormalities in either the blastocyst or the ZP, may be represent one of the factors causing implantation failure [1].

**Fig. 1:**
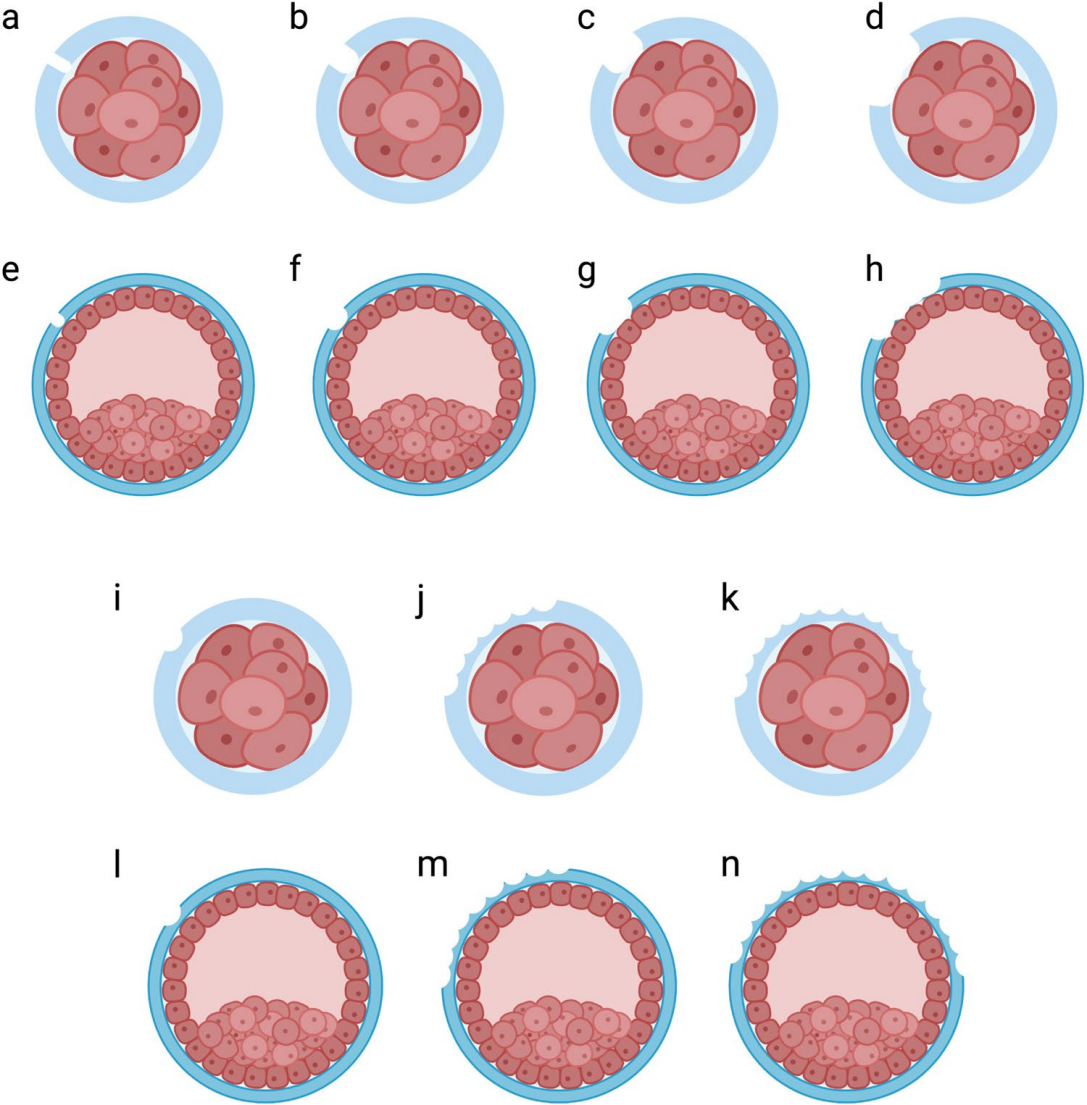
Procedures that are commonly used for assisted hatching (AH). The zona pellucida of embryos at the cleavage or blastocyst stage can be completely drilled with a small opening (< 10 µm (**a** and **e**) or 15 µm (**b** and **f**)) or a larger opening (25 µm (**c** and **g**) or > 25 µm (**d** and **h**)). Alternatively, the zona pellucida of embryos at the cleavage or blastocyst stage can be thinned at one point (**i** and **l**), continuing for one quarter (**j** and **m**), or half of the zona pellucida (**k** and **n**)

The goal of assisted hatching (AH) is to create a weakness on the ZP, thus helping the embryo to hatch and maximising the chance of implantation. ZP drilling/breaching was the first AH method reported by Cohen et al., who used mechanical force to create a gap in the ZP of embryo [1]. Since then, several procedures have emerged; these show significant variability in terms of methodology, the stage of embryo development, and the groups of patients in which AH is deployed [2]. For instance, ZP thinning is proceeded by using chemical solution or laser beam to digest or ablate the ZP partially, creating different extension [2]. However, after more than 25 years of application, there is still no standardised protocol for AH. The figure below illustrates the commonly used procedure for embryo AH (Fig. 1). In addition, much of the published data relating to the efficacy of AH are controversial, inconclusive, or confounded by variables related to differing techniques and methods that could not be controlled in a reliable manner [3–5]. Although, there has been much effort to study the association between different AH techniques and pregnancy outcomes [6–10], there has been little consensus regarding evidence-based guidelines with which to optimise AH protocols. In addition, whilst systematic reviews and guidelines relating to AH have described the overall application of AH [11–15], there is a significant paucity of data relating to the current deployment of AH in different fertility centres. The purpose of this study was to investigate the specific manner in which AH is applied in current fertility centres. Herein, we attempted to describe the different AH methodologies being used to perform AH and to identify potential opportunities for standardisation.

## Methods

Based on an extensive literature search and by considering expert opinions, we developed a novel questionnaire referred to as “The application of assisted hatching in IVF centres”. The questions focused on (1) clinical demographics: the location and treatments provided, and whether the centre is privately or publicly funded; (2) the application of AH prior to embryo biopsy (AHpBP) and AH prior to embryo transfer (AHpET); and (3) strategies of AH, including clinical indications, frozen/thawed or fresh embryos, stage of embryo development, embryo culture, and embryo-transfer strategies, as well as detailed information relating to AH techniques, protocols ( ZP drilling or ZP thinning), the size of the ZP opening, and the extension of thinning. A text box was provided after each question for responding clinics to provide their own comments (The questionnaire was provided in the supplementary material).

This anonymous questionnaire was conducted through an online survey platform (SurveyMonkey Inc., San Mateo, CA, USA; www.surveymonkey.com). The survey links were circulated through newsletters and email by appropriate learned societies devoted to embryologists and fertility specialists, including the Human Fertility and Embryology Authority (HFEA), the Association of Clinical Embryologists (ACE), and the Society for Reproduction and Fertility (SRF). No reminders were sent, given the intention to ensure anonymity. Ethical approval was not required because this was an anonymous survey and did not involve any patient interventions or the collection of personal data. The online survey was open for responses between October 2019 and March 2020. If there were two responses from one fertility centre, then these duplicated results were discarded; we also discarded responses with an incomplete dataset. The Chi-squared (*χ*^2^) test or Fisher’s exact test was used to analyse the frequency distribution of categorical variables and application rates amongst fertility centres within the UK and outside of the UK.

## Results

### Demographics

Table 1 outlines demographic and clinic data. A total of 129 fertility centres responded to the survey. These centres were located in 17 countries/regions, including China (52.2%, 66), UK (32.6%, 42), and other countries (16.3%), including Argentina, Cambodia, Canada, Cyprus, Denmark, Egypt, Greece, India, Italy, Malaysia, New Zealand, Norway, Qatar, Spain, and the USA. Analysis showed that 87.5% (113/129) of the responding fertility centres were able to provide advanced ART treatments, such as in vitro fertilisation (IVF) and intracytoplasmic sperm injection (ICSI); moreover, 46.9% (53/113) of these centres were able to conduct embryo biopsy for preimplantation genetic testing (PGT). In addition, 12.4% (16/129) of the responding fertility clinics stated that they only provided intrauterine insemination (IUI) treatment (Table 1). Only centres who administered advanced ART treatment were asked to provide further details with regards to AH.

**Table 1:** The demographics of responders

Demographics	*n*	%
Clinic location		
United Kingdom	42	32.6
Countries within the EU	8	6.2
Countries outside the EU	79	61.2
Type of clinics		
Publicly funded clinics providing publicly funded cycles	21	16.3
Publicly funded clinics providing privately/publicly funded cycles	57	44.2
Private clinics providing privately and publicly funded cycles	24	18.6
Private clinics providing privately funded cycles	27	20.9
ART treatment provided by clinics		
Advanced ART treatment, e.g. IVF, ICSI, and PGT	113	87.6
Only basic fertility treatment, e.g. ovulation induction and IUI	16	12.4
PGT (embryo biopsy) available in clinic?		
No	60	53.1
Yes	53	46.9

*EU* European Union, *ART* assisted reproductive technology, *IVF* in vitro fertilisation, *ICSI* intracytoplasmic sperm injection, *PGT* preimplantation genetic testing, *IUI* intrauterine insemination

### The Application of AH

**Fig. 2:**
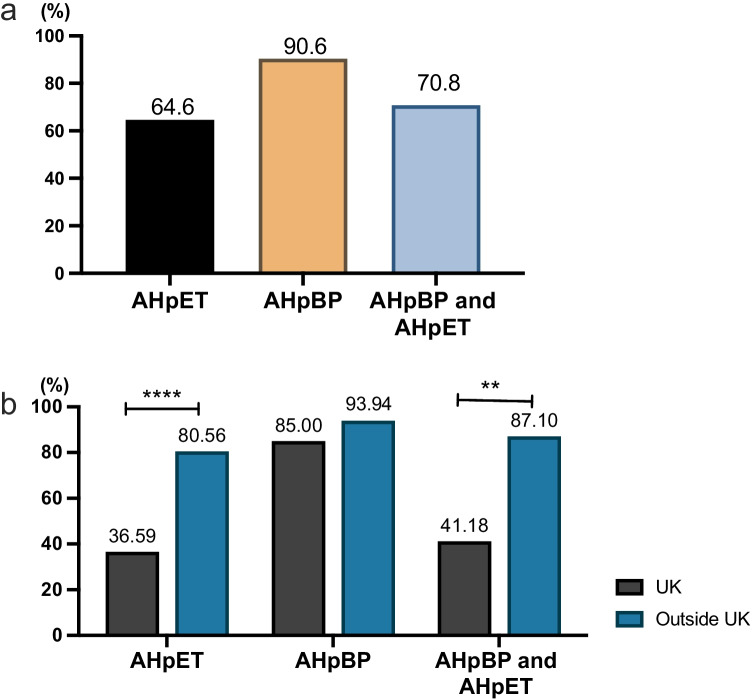
The application rate of assisted hatching (AH). **a** The application rates of AH prior to embryo transfer (AHpET), AH prior to biopsy (AHpBP), and both AHpET and AHpBP. **b** A comparison of application rates between regions outside of the UK and in the UK. The Chi-squared (*χ*^2^) test was used to compare the frequencies between two groups (**P < 0.01, ****P < 0.0001)

To separate AH by specific application, AH can be classified as AH prior to embryo transfer (ET) (AHpET) and AH prior to biopsy (AHpBP). AHpET was carried out in 64.6% (73/113) of centres administering advanced ART treatments, whilst 90.6% (48/53) of centres providing PGT treatment performed AHpBP (Fig. 2a). Furthermore, AHpBP was used prior to trophectoderm biopsy in 92.2% (47/51) of centres whilst 70.8% (34/48) of centres stated that they would administer both AHpBP and AHpET. Intriguingly, the frequencies of AHpET (36.6% vs 80.6%, *P* < 0.0001) and AH prior to both ET and biopsy (41.2% vs 87.1%, *P* = 0.002) were significantly lower in the UK when compared to those in centres from other countries; there was no significant difference with regards to the application of AHpBP (84.2% vs 93.9%) (Fig. 2b).

### The Application of Different Forms of AH

#### The Practice of AHpBP

The clinical practice of AHpBP is summarised in Table 2. Of the 48 centres that provided AHpBP, the time point for performing AH showed variability: 54.2% stated that they performed AH on the day before biopsy whilst 45.8% performed AH on the day of biopsy; some even performed AH immediately (less than 1 h) before biopsy (16.7%). With regards to the stage of embryonic development, 31.3% of centres stated that they performed AHpBP on day 3 embryos, 20.8% on day 4 embryos, whilst 41.7% performed AH at the blastocyst stage. With regards to the blastocyst biopsy procedure, 92.2% (47/51) of centres stated that they would perform AHpBP, with 55.3% (26/47) of them performed on the day before TE biopsy. To be specific, 53.8% (14/26) of AHpBP procedures for TE biopsy were performed on day 3 and 38.5% (10/26) were performed on day 4. The remaining centres stated that they would perform AHpBP on the day of TE biopsy (43.8% [21/48]) or immediately prior to TE biopsy (14.6% [7/48]).

**Table 2:** The characteristics of AHpBP practice

Characteristics/ details	*n*	%
Embryo stage for biopsy		
Day 3 embryos	2	3.8
Blastocyst embryos	36	67.9
Both of day 3 and blastocyst embryos	15	28.3
The time of AHpBP prior to biopsy		
Before the day of biopsy	26	54.2
On the day of biopsy	22	45.8
Embryo stage for AHpBP		
Cleavage stage (day 2)	1	2.1
Cleavage stage (day 3)	15	31.3
Morula stage (day 4)	10	20.8
Blastocyst stage (days 5–7)	20	41.7
Day 3 (cleavage) or days 5–7 (blastocyst)	2	4.2
Technique for AHpBP		
Laser-assisted method	46	95.8
Chemical method	1	2.1
Mechanical method	1	2.1
Method for AHpBP		
ZP drilling	38	79.2
ZP thinning	5	10.4
Both	5	10.4
The opening size of drilling (6 skipped the question)		
< 10 µm	14	37.8
10–15 µm	12	32.4
15–25 µm	9	24.3
> 25 µm	2	5.4
The extension of ZP thinning		
< Quarter of the circumference	7	70
Quarter of the circumference	1	10
Quarter to half the circumference	1	10
Half the circumference	0	0
> Half the circumference	1	10

*AHpBP* assisted hatching prior to biopsy, *ZP* zona pellucida

Analysis showed that laser-assisted AH was the most predominant technique (95.8%) and that ZP drilling was the main technique used for AHpBP (79.2%). However, the data relating to AHpBP drilling were inconsistent:  70.2% of centres stated that they would create a small ZP opening (< 15 µm); 24.3% centres stated that they would drill the ZP with an opening of 15–25 µm, and only 5.4% would drill an opening in the ZP that was > 25 µm in size. Moreover, 20.8% (10/48) of centres stated that they performed ZP thinning for AHpBP whilst 70% of them ablated less than one quarter of the ZP circumference.

#### The Practice of AHpET

With regards to the clinical indications for AHpET treatment, we found that ‘embryos with a thick ZP’, ‘patients with a poor prognosis’, and ‘frozen/thawed embryos’ were reported with similar levels of importance, thus accounting for 34.7%, 32%, and 30.6% of centres. Two centres claimed that AHpET was performed upon patient request whilst two other centres confirmed that AHpET was applied to all embryos. In addition, in 59.2% of the responding centres, AHpET was performed on both fresh and frozen/thawed embryos. In contrast, only 36.7% of centres applied AHpET on vitrified/warm and/or slow freeze/thaw embryos (Table 3).

**Table 3:** The characteristics of AHpET practice

Characteristics/ details	*n*	%
Indications for AHpET		
Embryos from patients with a poor prognosis	47	32.0
Embryos with a thick zona pellucida	51	34.7
Slow frozen/thaw or vitrified/warm embryos	45	30.6
Only when patient requests	2	1.4
All embryos	2	1.4
Fresh or frozen/thaw embryos		
Fresh embryos	3	4.2
Vitrified/warm embryo only	20	28.2
Slow freeze/ thaw embryo and/or vitrified/warm embryo	6	8.5
All embryos	42	59.2
Embryo stage for AHpET		
Cleavage stage (day 2)	6	5.6
Cleavage stage (day 3)	50	46.3
Morula stage (day 4)	6	5.6
Blastocyst stage (days 5–7)	46	42.6
Technique for AHpET		
Laser-assisted method	68	95.8
Chemical method	1	1.4
Mechanical method	2	2.8
Method for AHpET		
ZP drilling	28	39.4
ZP thinning	29	40.8
Both	14	19.7
The opening size of drilling (13 skipped this question)		
< 10 µm	12	40
10–15 µm	9	30
15–25 µm	5	16.7
> 25 µm	4	13.3
The extension of ZP thinning (5 skipped this question)		
< Quarter of the circumference	14	36.8
Quarter of the circumference	16	42.1
Quarter to half the circumference	8	21.1
Half the circumference	0	0
> Half the circumference	0	0
Continuously culture embryo after AHpET (3 skipped this question)		
Yes, culture to blastocyst stage	4	5.7
No, embryo(s) will be transferred very soon (within few hours)	43	61.4
Both, depends on embryo development or biopsy needs	23	32.9

*AHpET* assisted hatching prior to embryo transfer, *ZP* zona pellucida

With regards to AHpET, 46.3% of centres stated that they performed AH on day 3 embryos whilst 42.6% of centres stated that they performed this procedure on day 5–7 embryos (blastocyst stage). Laser-assisted AH was the main technique deployed; however, the methods used for ZP manipulation showed wide variation; 39.4% of centres stated that they would perform ZP drilling, 40.8% stated that they would use ZP thinning, and 19.7% stated that they use both methods. With regards to ZP drilling, 70% of centres stated that they would create a small opening in the ZP (< 15 µm), including 81.8% (18/22) of cleavage stage AHpET, and 61.9% (13/21) of blastocyst stage AHpET. In contrast, only 30% of centres stated that they would create an opening larger than 15 µm in the ZP. To be specific, an opening larger than 15 µm would be created in 18.2% (4/22) of centres performing AHpET at the cleavage stage and in 38.1% (8/21) of centres performing AHpET at the blastocyst stage. With regards to the extension of ZP thinning, 42.1% of centres stated that they would ablate one quarter of the ZP circumference whilst 36.8% stated that they would thin less than one quarter of the ZP circumference.

When asked whether they continued to culture embryos after AH, most of the centres (61.4%) stated that they would transfer the embryo within a few hours without extending the period of embryo culture, no matter whether AHpET was conducted at the pre-blastocyst stage (58.2% [32/55]) or the blastocyst stage (65.2% [30/46]). In contrast, only 5.7% of centres stated that they would culture embryos to the blastocyst stage (Table 3).

With regards to the cost of AH, 46.4% (39/84) of centres confirmed that they would charge for all AH procedures, whilst 5.9% (5/84) stated that they would only charge for AHpBP; 10.7% (9/84) of centres said that they would charge for AHpET. However, there were 36.9% (31/84) of centres claiming that they did not charge for AH.

With regards to the centres that did not apply AHpET, 64.1% (25/39) stated that that they did not perform this technique because there is no specific evidence for the efficacy of AHpET. Two centres from the UK highlighted that AHpET was considered as a red ‘add on’ on the HFEA’s traffic light system. In addition, 28.2% of centres stated that they did not perform this technique due to a lack of equipment; 15.4% of centres stated that they were cautious about this procedure because of safety concerns.

## Discussion

The contradictory efficacy of AH reported in the literature may be associated with the variety of AH procedures used in clinical practice. It is of significant concern that there is no standardised protocol or guidelines that regulate the application of AH. To the best of our knowledge, this is the first and most extensive investigation of data related to the current clinical practice of AH across different fertility centres. The most recent national summary of AH was released by the Centres for Disease Control and Prevention and showed that the utilisation of AH in the USA had increased significantly from 25,725 to 35,518 between 2000 and 2010 [4]. This increasing trend may relate to the guidelines published in 2008 by the American Society for Reproductive Medicine (ASRM) which suggested that AH may be clinically useful in patients with a poor prognosis [16]. Although no updated global registry is available to reflect the exact application rate of AH, according to the results in this study, it appears that AH is still being actively widely used in ART cycles, especially prior to embryo biopsy (AHpBP) (by more than 90%). Compared to AHpBP, AHpET is not so widely accepted, especially in the UK (36.6%). Intriguingly, in 2001, a multi-centre survey involving HFEA-listed clinics revealed that AH is performed widely (65%) in the UK [17]. This change in the UK is highly likely to be due to the National Institute for Health and Care Excellence (NICE) guideline update that was published in 2017, which stated that AHpET is not recommended as it has not been shown to improve pregnancy rates [18]. As demonstrated in the present study, the main reason that some centres do not apply AHpET is the lack of basic evidence for the efficacy of AH. Therefore, the application rate for AHpET in UK fertility centres is much lower than in those from outside the UK.

It is commonly agreed that AHpET should be applied to patients with specific clinical indicators rather than to a universal patient population. This study identified specific indicators for AHpET with equal importance: embryos from patients with a poor prognosis, embryos with a thick ZP, and frozen/thawed embryos. The 2008 ASRM guidelines [16] stated that a poor prognosis was a clinical indicator for AH; this was further supported by the publication of data showing that AH improved the rates of both pregnancy and implantation [19, 20]. However, in 2014, the ASRM updated their guidelines to state that it is premature to recommend AH for all patients with a poor prognosis [12] because of insufficient clinical evidence [4, 13]. However, it is noteworthy that different clinics may use different sub-groups of patients with a poor prognosis as the indication for AH; this may partially explain the inconsistency of AH efficacy in this particular patient group. For instance, a meta-analysis of randomised clinical trials (RCTs) conducted by Wellington [15] found that AH could only improve the clinical pregnancy rate and live birth rate in women who had experienced previous repeated implantation failure, rather than women of advanced age. Furthermore, a recent study also suggested that repeated implantation failure alone is not an indicator for AH, and that AH may hamper implantation in younger patients [21]. On the other hand, it is understandable that patients with a poor prognosis may seek all possible medical intervention to achieve pregnancy. Thus, until we have robust and validated evidence, clinics may still apply AH in ART cycles to fulfil the psychological needs of their patients after fully informing the patients of the potential risks and chances of success.

Aside from patients with a poor prognosis, the other important indications for AH were frozen/thawed embryos and embryos with a thick ZP. Previous research suggested that the cryopreservation process could cause abnormalities in the ZP, such as hardening and thickening, thus leading to hatching difficulties [22]. Indeed, AH has been demonstrated to improve the pregnancy outcomes of frozen/thawed embryos at different stages of embryonic development [15, 23–26]. However, a previous meta-analysis reviewed 12 RCTs and found that when performed on cryopreserved-thawed embryos, AH was correlated with a higher clinical pregnancy rate and implantation rate but had little effect on live birth rate [27]. More recent studies showed that AH prior to transfer was not associated with improved pregnancy outcomes when applied on frozen/thawed embryos [28, 29]. Furthermore, some studies even reported reduced live birth rates following AH in FET cycles [30, 31]. However, AH approaches, embryo stages, and embryo quality all represent potentially confounding factors; furthermore, detailed information relating to the specific procedure used for AH was lacking in some studies. Therefore, a more comprehensive evaluation of the use of AH in frozen/thawed embryos and different patient groups is very important.

The application of infra-red lasers has become the predominant technique for both AHpBP and AHpET in clinical practice. However, the present study also identified an extensive disparity in the practice of AH. Traditionally, AHpET is mostly widely performed at the cleavage stage [32]. However, in the present study, 42.6% of centres preformed AHpET at the blastocyst stage. The increasing trend to perform AHpET at the blastocyst stage is likely to be due to the high precision and control provided by lasers, thus making it easier to perform AH at this stage. Although several studies have focused on the outcomes of AH at the blastocyst stage [24, 28, 29, 33–35], current evidence remains inconsistent and inconclusive. In addition, no study to date has investigated the effect of this procedure on different embryo development stages. Therefore, studies focusing on the correlation between different AHpET timepoints and outcomes may be very informative for the future evaluation of efficacy.

Furthermore, there is no consensus with regards to the timing of embryo transfer following AHpET. We found that most centres prefer to transfer embryos within a few hours of AH rather than culturing them to the blastocyst stage. Meanwhile, several lines of evidence have suggested that blastocyst transfer is the more desirable choice because of the extended culture duration and the consequential improvement in implantation [36, 37]. Therefore, continuing embryo culture to the blastocyst stage after AH could be beneficial. In addition, a recent study reported that AH may help lower grades of cleavage stage embryo to develop to usable blastocysts [38]. However, the evaluation of AH in day 3 embryos followed by blastocyst transfer is exceptionally sparse [39, 40]. Therefore, future research is needed to understand the effect of extending the duration of culture following AH.

Although trophectoderm (TE) biopsy has become the dominant method for biopsy, the procedure used for AHpBP shows significant variation in clinical practice and there is no standard protocol recommended by current guidelines. According to the best practice guideline out forward by the ESHRE consortium, ZP drilling for TE biopsy can be performed on day 3 or the morning of day 5, followed by the removal of TE cells [41]. This was clearly apparent in our data; apart from day 3 and blastocyst stage, 38.5% of AHpBP procedures for TE biopsy were performed on day 4. This was in slight disagreement with the current recommendations. However, so far, there has been a significant lack of research with regards to the effect of different AHpBP timepoints on subsequent TE biopsy procedures and pregnancy outcomes. Only one recent study compared the day 3 prehatching protocol (AH on day 3 and biopsy at the blastocyst stage) and sequential hatching and biopsy protocol (AH and biopsy at the blastocyst stage); the authors involved in this study found that the pregnancy outcomes were significantly better when using the sequential hatching and blastocyst biopsy protocol [42]. In addition, it has been demonstrated that pre-hatching at the cleavage stage could potentially increase the risk of ICM incarceration and may require extra manipulation during biopsy [43]. Therefore, it is very important to comprehensively investigate the potential effect of different AH protocols on TE biopsy procedures and subsequent pregnancy outcomes.

Another wide variation was found as regards the details of AH handling and the invasive extension of ZP manipulation. Our survey did demonstrate that ZP drilling was far more extensively used for AHpBP (79.2%), whilst ZP drilling and ZP thinning accounted for similar proportions of all AHpET procedures (39.4% vs 39.4%). As discussed in a recent systematic review, even when only using the laser-mediated approach, it is unclear whether embryos benefit from ZP drilling or thinning [2]. Furthermore, some studies have attempted to compare ZP drilling and ZP thinning; nevertheless, discrepancies in study power and the variation in AH technique made it difficult to define a better method [10, 44–47].

Although there is no universal protocol for ZP drilling or ZP thinning, it is important to note that the invasive extension of ZP manipulation plays a significant role on the outcome of AH. In this study, the extension of ZP thinning was consistent in that most centres would ablate a ZP with less or equal to one quarter of the ZP circumference. On the other hand, the majority of centres stated that they drill the ZP with a small opening (< 15 μm), regardless of whether they are performing AHpBP or AHpET. Although a significant relationship between the size of ZP opening and the in vitro hatching has been described in many animal studies [48–50], sparse attention has been paid to the correlation between the size of the hole drilled and embryo hatching outcomes in the clinic, since embryos would only be kept in culture for a short time to the blastocyst stage if not transferred very soon after AH. On the other hand, the efficacy of the total removal of the ZP has been highlighted as none of the existing studies demonstrate that zona removal would reduce clinical outcomes [2]. However, according to this survey, very few centres applied AH with a highly invasive extension of ZP drilling or thinning. Therefore, more evidence is needed to verify the benefit of invasive extension of ZP manipulation, such as total removal of the ZP, before applying this technique routinely.

Apart from the invasive extension associated with ZP manipulation, the possible effect of the site used for AH also needs to be considered. A previous study showed that the hatching process exhibited a degree of polarity in that the performing AH at a site close to the ICM resulted in a higher rate of complete hatching, whilst AH performed at the opposite site caused trapping of the ICM within the ZP [34]. However, Ren et al. found that the site used for AH has no influence on the rates of implantation, pregnancy, and live birth in human vitrified-warmed blastocysts [35]. The true value of the AH site on the blastocyst remains undetermined and requires further investigation.

As indicated from this study, owing to the considerable disparity in AH practice, the narrow patient inclusion criteria, and the complex nature of pregnancy, it is possible that the beneficial effects of AH may be masked in a well-defined patient population. Thus, the complete rejection of AH may seem rather unfair and premature. Whilst waiting for the next large-scale and rigorous RCTs to test the efficacy of AH, such as the trial currently being performed in Italy to investigate the efficacy of AH on vitrified/warmed blastocysts [51], it is recommended that researchers and clinical communities should work together, using biostatistics and data management techniques, to develop large prospective and well-phenotyped cohorts to comprehensively characterise AH and guide their application in clinical practice [52].

Our study is limited by the potential bias that exists in survey-based investigations; the centres included might not form a representative sample of world-wide IVF centres, since most responses were from UK and China. In addition, given the nature of voluntary surveys, some questions may have been skipped before submission. Thus, there were slight differences in the numbers of responses from question to question. Finally, to limit the length of the survey, some areas of AH were not included, such as the power and irradiation time of the laser applied, the mean length of time that the embryo remained outside of the incubator, the site of drilling/thinning, and the depth of ZP ablation.

## Conclusion

Our analyses confirmed that AH is still widely used, especially prior to biopsy. However, there is an extensive disparity in the practice of AH, this leading to uncertainties and inconsistencies when reviewing the literature. Such inconsistency highlights the need for protocol standardisation to reduce the variation that clearly occurs in clinical practice. This will finally allow us to evaluate the efficacy and safety of AH in a comprehensive manner. Finally, there is a clear need for a multi-centre, large, prospective, and carefully phenotyped cohort study, and RCTs, to fully understand the efficacy and safety of AH in clinic practice.

## Supplementary Information

Below is the link to the electronic supplementary material.

Supplementary file1 (DOCX 31 KB)
