# Supplementary material for: The Current Practice of Assisted Hatching for Embryos in Fertility Centres: a General Survey
Source: Reprod Sci. 2022 Apr 11;29(9):2664–73. doi: 10.1007/s43032-022-00931-0 (PMC9444829; doi:10.1007/s43032-022-00931-0)
Supplement: Supplementary file 1 — Supplementary file1 (DOCX 31 KB) [file 43032_2022_931_MOESM1_ESM.docx]

# **The application of assisted hatching in IVF centres**

1. What is your job title?

- Clinician
- Embryologist
- Nurse
- Other, _____

1. What type of clinic are you working at?

- Publically funded, eg. NHS
- Private clinic, providing privately funded cycles only
- Private clinic – providing privately and publicly funded cycle
- Research
- Other, ____

1. What is the name of your clinic?

___________________

1. Where is your clinic (country)?

- UK
- Other EU countries (specifically), _____
- Outside EU (specifically), ______

1. Which treatments can your clinic provide?

- Basic fertility treatment, e.g. IUI
- Advanced fertility treatment e.g. IVF/ ICSI, PGT

1. Does your clinic perform PGT (embryo biopsy)?
   - Yes, Day 3 (cleavage stage) only

- Yes, blastocyst only
- Yes, all stages
- No

1. Does your clinic perform assisted hatching routinely before the biopsy (AHpBP)?
   - Yes, before the day of biopsy
   - Yes, on the day of biopsy
   - Yes, immediately before biopsy (<1hr)
   - No
2. Which day do you perform assisted hatching prior to biopsy (AHpBP)?
   - Cleavage stage-Day 2
   - Cleavage stage-Day 3
   - Morula stage-Day4
   - Blastocyst stage-Day 5-7
3. What technique do you use for assisted hatching prior to biopsy (AHpBP)?

- Laser method
- Chemical method
- Mechanical method
- Don't know

1. Which process do you use to perform assisted hatching prior to biopsy (AHpBP)?

- Zona pellucida drilling/Opening
- Zona pellucida thinning
- Both
- Don't know
- Comment, ______

1. If drilling, what is the size of the opening?

- Less than 10μM
- 10-15 μM
- 15-25μM
- More than 25μM
- Don't know
- Comment, _____

1. If thinning, what is the extension of ZP ablation?

- Less than a quarter of the circumference
- A quarter of the circumference
- A quarter to a half of the circumference
- Half of the circumference
- More than half of the circumference
- Don't know
- Comment, ____

1. Does your clinic use assisted hatching without performing biopsy, but prior to embryo transfer (AHpET)?
   - Yes
   - No
2. If No AHpET, what is the reason?
   - - Unsure of efficiency
     - Unsure of safety
     - No equipment
     - No needed
3. If Yes, in which cases do you use AHpET? (Please tick all that apply

- All embryos.
- Embryos from patients with poor prognosis, such as recurrent implantation failure and advanced age.
- The embryos with a thick zona pellucida.
- Slow frozen/thaw or vitrified/warm embryos
- Comment, ______

1. Which stage of embryos are treated with AHpET?
   - Cleavage stage (Day 2)
   - Cleavage stage (Day 3)
   - Morula stage (Day 4)
   - Blastocyst stage (Day 5-7)

- Don't know

1. What technique do you use for AHpET?

- Laser method
- Chemical method
- Mechanical method
- Don't know

1. Which process do you use to perform AHpET?

- Zona pellucida drilling/Opening
- Zona pellucida thinning
- Both
- Don't know
- Comment, ______

1. If drilling, what is the size of the opening?

- Less than 10μM
- 10-15 μM
- 15-25μM
- More than 25μM
- Don't know
- Comment, _____

1. If thinning, what is the extension of ZP ablation?

- Less than a quarter of the circumference
- A quarter of the circumference
- A quarter to a half of the circumference
- Half of the circumference
- More than half of the circumference
- Don't know
- Comment, ____

1. Do you continue embryo culture following AHpET?
   - No, embryo(s) will be transferred very soon (within few hours).
   - Yes, the embryo(s) will be cultured to blastocyst stage.
   - Both, depends on embryo development or biopsy needs.
   - Don't know
2. Do you treat fresh or frozen-thawed embryos with pre-transfer assisted hatching without biopsy? (Please tick all that apply)
   - Fresh embryos

- Slow freeze/thaw embryos
- Vitrified/warm embryos
- Both

1. If you perform assisted hatching to cryopreserved embryos post-thaw/warm, what is the reason?

- ___________________

1. Do you charge extra for assisted hatching procedure and if so which scenario would you charge?

- No
- Yes, for biopsy only
- Yes, for improved outcome
- Yes, for all AH procedures

1. If you do not use AHpET nor AHpBP, what is the reason?
   - - Unsure of efficiency
     - Unsure of safety
     - No equipment
     - No needed
